# Healing the Sick

**Published:** 1887-10-15

**Authors:** 


### Healing the Sick.

By the Right Rev. the Lord Bishop of London.

It is nothing new to plead for the hospitals, because hospitals are old Christian institutions, and have been from very early days one of the marks of Christianity in any country. There is hardly any institution we can name which more emphatically appeals to Christian feelings, because there is hardly any other which so enables Christians to follow the example of our Lord and Master in relieving pain and removing the friction and disability caused by illness. We take our Lord as our model, and of all things which He did when on earth the healing of the sick was one of the most characteristic. It is true that we cannot work miracles, but, nevertheless, He, as being the source of all light, is the true source of our power to do this great service to our fellow-men ; and in doing it we feel we are doing what He would be doing, were He on earth, to relieve the sick of their pain. What a blessing it is to be in some degree able to mitigate suffering, though that is not all nor anything like all. Is there any charity which can be put by the side of that of restoring to a poor man the power of again working for his family and others who are dependent upon him, sending him back once more with health and strength to discharge the duties devolving upon him? Is there any blessing you can compare with that, and say that this healing of the sick must stand second, that something else must stand first? Certainly not, if we look to the example of the Lord Himself. Is there anything which teaches a man so much, which enables a man to lift up his heart in gratitude to God more effectually than when you have succeeded in restoring him to health and home? The hospitals do not preach from the pulpit, but they preach from the sick-bed ; they preach by the power of their work. They make people think more than anything else what it is to live in a Christian land and what it is to recognise Christian principles. Those who are supporting the hospitals are doing as much for the spread of the Gospel as can be done by many servants and much teaching, even though the servants are most powerful and the teaching most effectual. I do not deny that the work of the clergy is, in its own essence, a higher kind of work. The teaching of the Gospel is something higher and nobler than the healing of the sick, but the healing of the sick is the necessary accompaniment of the other ; and if you want to teach the Gospel effectually you cannot do it better than by helping the sick.

The population of this great city of London is spreading on all sides, and the few hospitals which have been set up, some of them centuries ago, are not sufficient to do the work which is required at the present day. We want more of the ancient munificence; and not only do we want that munificence which is given in large sums by those who can spare much, but we want the contributions of those who from day to day are able to give something towards this purpose and who can witness the effects of their gifts. We want more hospitals instead of fewer. I do not know that there is any other call that I shall ever dream of putting above this call of the hospitals. There is need to advocate the cause because the greatness of the necessity is not understood, people do not consider how it presses upon the classes who need it. It is not from any hardness of heart that they do not consider it, but because of their many occupations. We desire to awaken them and make all men understand why it is we are asking for money. Not only do the hospitals confer a very great blessing upon the generation that now exists and in which we are working, but they are conferring untold blessings upon generations to follow by giving those opportunities which they do for learning more and more of that wonderful work of God—the human body. What a marvellous blessing is the work of these hospitals, a blessing which many of us have shared already! How many a man has had his own pain alleviated by the skill of some physician, how many a man has had years added to his life by the exercise of that faculty which God has given us, and given us the means of using! All this comes to a large extent from hospitals. They are a double blessing ; a blessing to those who receive their actual assistance, and a blessing to the wealthier classes. The most skilful physician whom the rich may summon has had the advantage of studying in hospitals all forms of disease, and brings his knowledge to bear upon their particular cases. The wealthy are sharers in the blessings, perhaps even more than their poorer neighbours. Will they then not show their gratitude for this? Will they not show some gratitude by taking their part in supporting such work as this to the utmost of their ability?

